# Molecular signatures of labor and nonlabor myometrium with parsimonious classification from 2 calcium transporter genes

**DOI:** 10.1172/jci.insight.148425

**Published:** 2021-06-08

**Authors:** William E. Ackerman, Catalin S. Buhimschi, Ali Snedden, Taryn L. Summerfield, Guomao Zhao, Irina A. Buhimschi

**Affiliations:** 1 Department of Obstetrics and Gynecology, University of Illinois at Chicago College of Medicine, Chicago, Illinois, USA.; 2 The High Performance Computing Facility, The Abigail Wexner Research Institute at Nationwide Children’s Hospital, Nationwide Children’s Hospital, Columbus, Ohio, USA.; 3 The Ohio State University College of Medicine, Department of Obstetrics & Gynecology, Columbus, Ohio, USA.

**Keywords:** Reproductive Biology, Expression profiling

## Abstract

Clinical phenotyping of term and preterm labor is imprecise, and disagreement persists on categorization relative to underlying pathobiology, which remains poorly understood. We performed RNA sequencing (RNA-seq) of 31 specimens of human uterine myometrium from 10 term and 21 preterm cesarean deliveries with rich clinical context information. A molecular signature of 4814 transcripts stratified myometrial samples into quiescent (Q) and nonquiescent (NQ) phenotypes, independent of gestational age and incision site. Similar stratifications were achieved using expressed genes in Ca^2+^ signaling and TGF-β pathways. For maximal parsimony, we evaluated the expression of just 2 Ca^2+^ transporter genes, *ATP2B4* (encoding PMCA4) and *ATP2A2* (coding for SERCA2), and we found that their ratio reliably distinguished NQ and Q specimens in the current study, and also in 2 publicly available RNA-seq data sets (GSE50599 and GSE80172), with an overall AUC of 0.94. Cross-validation of the *ATP2B4*/*ATP2A2* ratio by quantitative PCR in an expanded cohort (by 11 additional specimens) achieved complete separation (AUC of 1.00) of NQ versus Q specimens. While providing additional insight into the associations between clinical features of term and preterm labor and myometrial gene expression, our study also offers a practical algorithm for unbiased classification of myometrial biopsies by their overall contractile program.

## Introduction

The denouement of physiological human pregnancy is heralded by the onset of parturition. Active term labor (TL) is diagnosed clinically by the presence of regular, forceful uterine contractions accompanied by progressive cervical remodeling (1). Therefore, by definition, successful normal labor requires synchronous changes in 2 separate regions of the uterus: the uterine corpus, which changes from a quiescent (Q) to a contractile phenotype, and the uterine cervix, which transitions from a long and closed configuration to an effaced and opened state. Although not part of the formal definition of labor, changes in the status of the fetal membranes are implied, as the amniochorion must rupture to allow for passage of the fetus and the placenta during the birthing process.

The above biological processes are clinically relevant because at term (>37 weeks of gestation), in the absence of functional labor, a cesarean delivery might be indicated. Additionally, term prelabor rupture of the membranes (PROM) unaccompanied by a smooth laboring process is also considered pathological, since it has the potential to increase risk for neonatal sepsis and maternal morbidity (2). Similarly, threatened preterm birth (PTB) may also vary in clinical presentation, and application of the criteria used for active TL can lack diagnostic precision (3–5). For example, while in some instances preterm labor may manifest with recognizable contractions and cervical changes, PTB may also follow isolated cervical shortening/dilation, uterine bleeding, or preterm PROM (PPROM) in the presence or absence of intraamniotic infection and/or inflammation (Triple I) (6, 7). However, even in the context of suspected or confirmed Triple I, patients may or may not present with the classical labor triad (i.e., progressive uterine contractility, cervical changes, and fetal membrane rupture) (8, 9).

It is reasonable to conclude that clinical phenotypic presentation may not always reflect activation of biological/functional genomic machineries responsible for myometrial contractility, cervical dilatation, or PPROM. This observation has led to spirited debates focused on resolving whether PTB occurring in the context of infection, inflammation, or bleeding should be classified as spontaneous or iatrogenic, even when uterine contractions are initiated by the health care provider (7, 10). Villar et al. proposed placing clinical chorioamnionitis, infection/fetal inflammatory response syndrome, and histological chorioamnionitis in distinct phenotypic categories (7). While this phenotypic classification categorized clinical chorioamnionitis together with preeclampsia-eclampsia, Manuck et al. placed infection/inflammation in the spontaneous PTB category (10). Such classification frameworks are valuable in their attempt to create consistency, but they are syndromic and lack precision related to mechanisms that would be important to devise prevention strategies.

The transition of the myometrium from functional quiescence to that of a contractile laboring state is a complex process characterized by multiple anatomical and molecular alterations that are incompletely understood and poorly defined. Uterine quiescence is maintained by numerous biological mediators (e.g., progesterone, prostacyclin, nitric oxide, and relaxin) that serve to limit the electrophysiological events that would otherwise drive coordinated smooth muscle contractility (11). Conversely, a confluence of complex molecular and electrical signaling events is required for uterine activation, including increased expression of gap junctions.

Several studies to date have attempted to characterize the transcriptional landscape of the myometrium to identify the gene sets involved in the initiation and/or maintenance of labor (12–16). The status of a specimen as labor or nonlabor is almost always appreciated clinically based on a well-recognized definition of labor, which includes both the uterus and cervix. The objectives of the current study were (a) to perform a transcriptomics study of a larger number of human myometrium specimens with detailed clinical phenotypic information to identify core sets of transcripts associated with the states of myometrial quiescence and contractility (nonquiescence); (b) to validate a practically feasible, parsimonious algorithm that distinguishes — at a molecular level — a contractile from a Q myometrium; and (c) to provide additional mechanistic insight in the pathogenesis of PTB by examining pathways and transcripts differentially regulated between the contractile and Q state.

## Results

### Clinical characteristics of myometrial sample donors.

**Figure 1:**
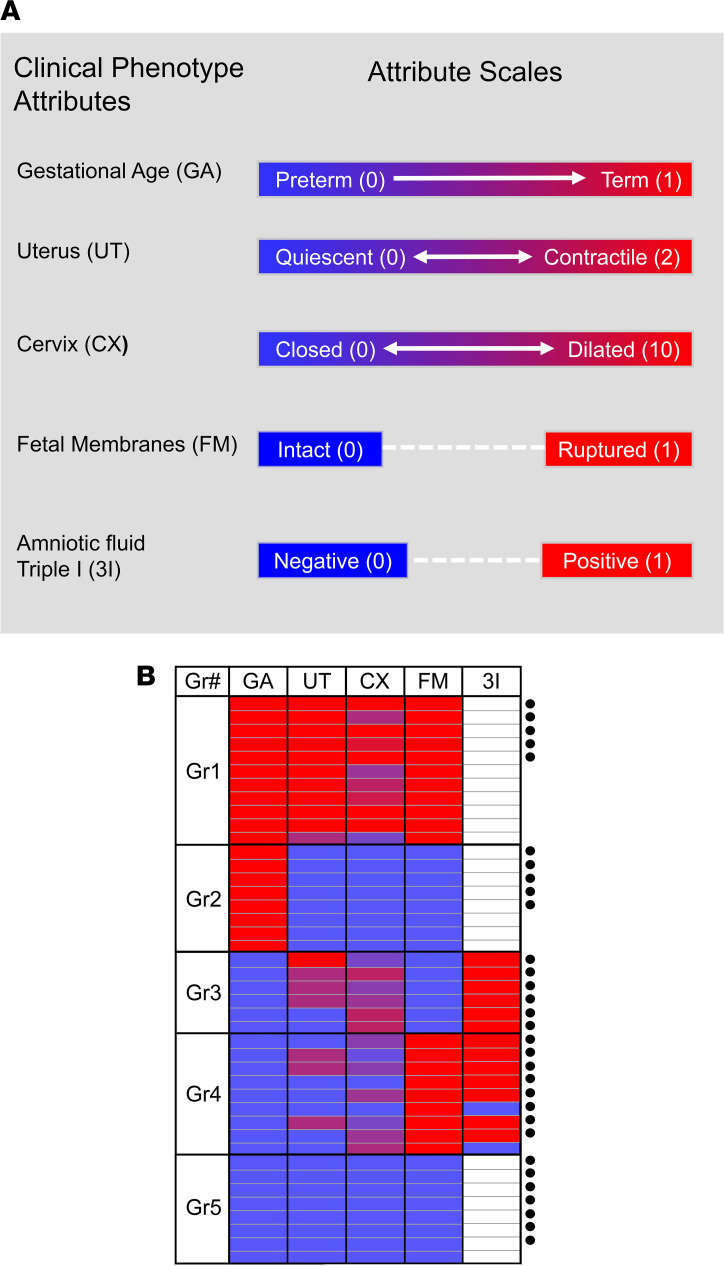
Overview of clinical characteristics of myometrial samples. (**A**) Graphical summary of clinical phenotypic attributes included as part of the classification algorithm used in this study. Gestational age was established clinically based on last menstrual period and/or ultrasonographic examination prior to 20 weeks (wk) of gestation and dichotomized into term (1, delivery > 37 wk of gestation) and preterm (0, delivery < 34 wk of gestation) cases. Uterine contractions were scored semiquantitatively on a scale from 0 to 2 (0, absent; 1, irregular, not followed by cervical change or when contractions receded after tocolysis; or 2, regular and followed by cervical change). Cervical dilation was scored on a scale from 0 to 10 cm as recorded on the last exam prior to cesarean. Membrane status was scored as 0 (intact) or 1 (ruptured). Triple I was scored as 0 (absent) or 1 (confirmed or suspected). (**B**) Heatmap of clinical characteristics for all cases included in the current study. Cases were aggregated into 5 groups as follows: Gr1, term birth following spontaneous onset of term labor (TL); Gr2, term birth by elective cesarean section not in labor (TNL); Gr3, PTB following spontaneous preterm labor with intact membranes (PTB-sPTL); Gr4, PTB following PPROM (PTB-PPROM); and Gr5, provider-initiated PTB in the absence of active labor contractions, cervical dilation, or membrane rupture (PTB-PI). Additional phenotyping of cases spontaneously committed to PTBs (Gr3 and Gr4) involved presence or absence of Triple I based on cultures of amniotic fluid obtained via clinically indicated amniocentesis as described in prior studies. The circular symbols to the right of the image denote samples that were included in the exploratory phase of the study.

A visual summary of the clinical characteristics included in our phenotyping algorithm is presented in Figure 1A. For the purpose of group analyses, cases were aggregated by gestational age (GA) and clinical characteristics as follows: Group 1: term birth following spontaneous onset of TL (*n* = 5; GA, 40 ± 1 week [mean ± SD]). Group 2: term birth by elective cesarean section not in labor (TNL; *n* = 5; GA, 39 ± 1 week). Group 3: PTB following spontaneous preterm labor with intact membranes (PTB-sPTL; *n* = 6; GA, 26 ± 2 weeks). Group 4: PTB following PPROM (PTB-PPROM; *n* = 8; GA, 29 ± 3 weeks). Group 5: provider-initiated PTB in the absence of active labor contractions, cervical dilation, or membrane rupture (PTB-PI; *n* = 7; 29 ± 3 weeks). Because we acknowledge the imprecise nature of clinical phenotyping, along with the practical impossibility of collecting human myometrium not impacted by clinical care protocols aimed at maximizing maternal and neonatal well-being, we are providing additional clinical characteristics for each case in Supplemental Table 1 (supplemental material available online with this article; https://doi.org/10.1172/jci.insight.148425DS1). The clinical characteristics associated with each case’s likelihood of progressing toward spontaneous labor and delivery were scored semiquantitatively, and Figure 1B presents a heatmap summarizing these characteristics for all the cases in the current study. Whereas Group 1 (TL) and Group 5 (PTB-PI) where clearly distinguishable, cases in Group 3 (PTB-sPTL) and Group 4 (PTB-PPROM) were in intermediate states based on uterine contractions and cervical dilatation.

The group characteristics for the cases that contributed to the RNA sequencing (RNA-seq) data sets is presented in Table 1. There were no statistically significant differences in GA (*P* = 0.740) or birth weight (*P* = 0.916) between the 2 term groups (TL versus TNL). Among PTB cases, spontaneous commitment to delivery was either through clinically confirmed PPROM (PTB-PPROM, cases MY11, MY14, MY15, MY23, MY25, MY26, MY28, and MY29), spontaneous preterm labor manifesting as either cervical shortening and dilation followed by onset of irregular uterine contractions (PTB-sPTL, cases MY13, MY24, MY27, MY30, and MY31), or by onset of uterine contractions accompanied by cervical change (PTB-sPTL, case MY12). Two cases in the PTB-PPROM group (MY25, MY28) had complex mixed phenotypes: in the first case (MY25), PPROM was followed by initiation of contractions that subsided following short-term tocolysis given to permit antepartum corticosteroid administration; in the second instance, uterine contractions that receded after tocolysis were followed by PPROM (MY28). Both patients were managed expectantly as PPROM until the diagnosis of Triple I (MY25) or fetal distress (MY28) prompted the surgical delivery where the myometrium was collected. PTB-PI cases (*n* = 7, MY16, MY17, MY18, MY19, MY20, MY21, and MY22) were all provider-initiated PTBs in the context of preeclampsia and/or eclampsia.

### Myometrial transcriptomics landscape of TL and projection of PTB cases.

**Figure 2:**
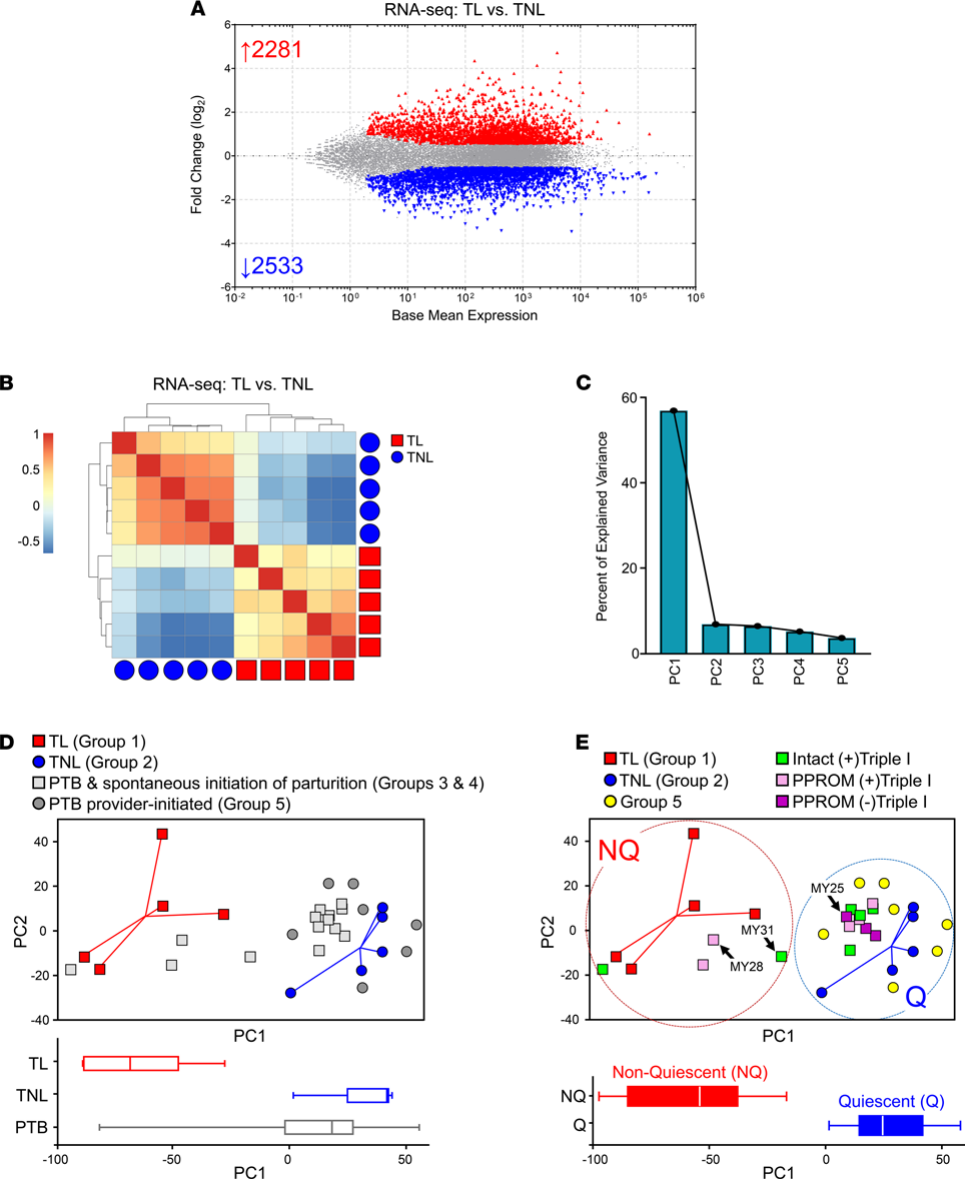
Differentially expressed RNA transcripts in myometrial specimens in term and preterm labor. (**A**) Plot of log_2_ ratio to average baseline (TNL) expression for the 4814 transcripts differentially abundant (FDR < 0.1, minimum fold-change ± 1.5, DESeq2 algorithm) between term myometrial specimens in the absence (TNL, *n* = 5) or presence (TL, *n* = 5) of labor. Transcripts with increased abundance are depicted in red, while those with decreased levels are shown in blue. (**B**) Correlation matrix with unsupervised hierarchical cluster analysis showing a higher degree of similarity among samples within each cohort (blue circles, TNL; red squares, TL) than between cohorts when considering the differentially abundant transcripts. (**C**) Scree plot of principal components following application of PCA to the differentially expressed genes in term myometrial specimens. The first principal component, PC1, accounted for most (57%) of the explained variance in the data. (**D**) Scatterplot of PC1 and PC2 with accompanying box-and-whisker plots (below) showing the distribution of TL (red squares), TNL (blue circles), and preterm birth (PTB, gray symbols) in PC space based on the expression signature of 4184 transcripts. Spontaneous initiation of PTB is denoted by light gray boxes, while dark gray circles indicate the absence of spontaneous labor initiation as determined clinically. (**E**) Scatterplot as in **D** but recolored to indicate in greater detail the clinical disposition of the pregnancies from which the PTB myometrial specimens were derived: intact membranes with Triple I (green squares); intact membranes without Triple I (Group 5, PTB-PI, yellow circles); preterm premature rupture of membranes (PPROM) with Triple I (pink squares); and PPROM without Triple I (purple squares). Two clusters comprising mostly nonquiescent (NQ) and quiescent (Q) myometrial specimens were evident (dashed circles). PTB cases with complex mixed phenotypes (MY25 and MY28), as well as a NQ specimen (MY31) that distributed between the term specimen clusters, are indicated.

RNA-seq analysis revealed extensive expression changes in TL myometrium compared with the TNL, with 4814 transcripts (21% of 22,630 detected transcripts) exhibiting statistically significant differences (absolute linear fold-change ≥ 1.5, FDR < 0.1; Figure 2A and Supplemental Table 2). Of these transcripts, 2281 were upregulated and 2533 were downregulated. Applied to this expression signature, unsupervised hierarchical clustering revealed a higher degree of positive correlation among samples within each clinical phenotype (TL or TNL) than between phenotypes with complete separation, as indicated by the dendogram in Figure 2B. Next, we performed principal component analysis (PCA) on the TNL and TL transcript expression data. As shown by the Scree plot in Figure 2C, the first principal component axis (PC1) accounted for 57% of the explained variance in the data. Figure 2D recapitulates the complete separation of the TL (red squares) and TNL (blue circles) specimens along PC1.

When the gene expression of PTB specimens was projected onto the PCA coordinates generated from TL and TNL, the preterm samples showed wide dispersion within PC coordinates and along PC1 (Figure 2D). A majority (17 of the 21 PTB specimens), including all provider-initiated PTB-PI (Group 5) samples, congregated near the TNL specimens. Only 4 of the 21 PTB samples clustered near the TL samples. Scrutiny of the detailed clinical records revealed that these 4 subjects manifested regular uterine contractions during hospitalization in close temporal proximity to the time of cesarean section. When PTB specimens were further grouped by Triple I status and fetal membrane integrity, it was noted that, among the 14 cases with spontaneous commitment to delivery (Group 3 and Group 4), 7 of the 11 (64%) samples from cases with Triple I diagnoses segregated with TNL samples (Figure 2D). Overall, cases could be separated based on PC1 into 2 molecular phenotype clusters: Q (*n* = 22) and nonquiescent (NQ, *n* = 9) — each inclusive of the TNL and TL cases, respectively (Figure 2E). Among all cases, PC1 correlated best with the score for contractions (Spearman’s *r* = –0.73, *P* < 0.001) followed by cervical dilatation (*r* = –0.68, *P* < 0.001) and membrane rupture (*r* = –0.52, *P* = 0.003). The complete list of the 4009 transcripts differentially abundant (absolute linear fold-change ≥ 1.5, FDR < 0.1) between PTB specimens exhibiting Q versus NQ myometrial phenotypes is presented in Supplemental Table 3.

### Characterization of the transcriptional signature associated with the myometrial NQ molecular phenotype.

**Figure 3:**
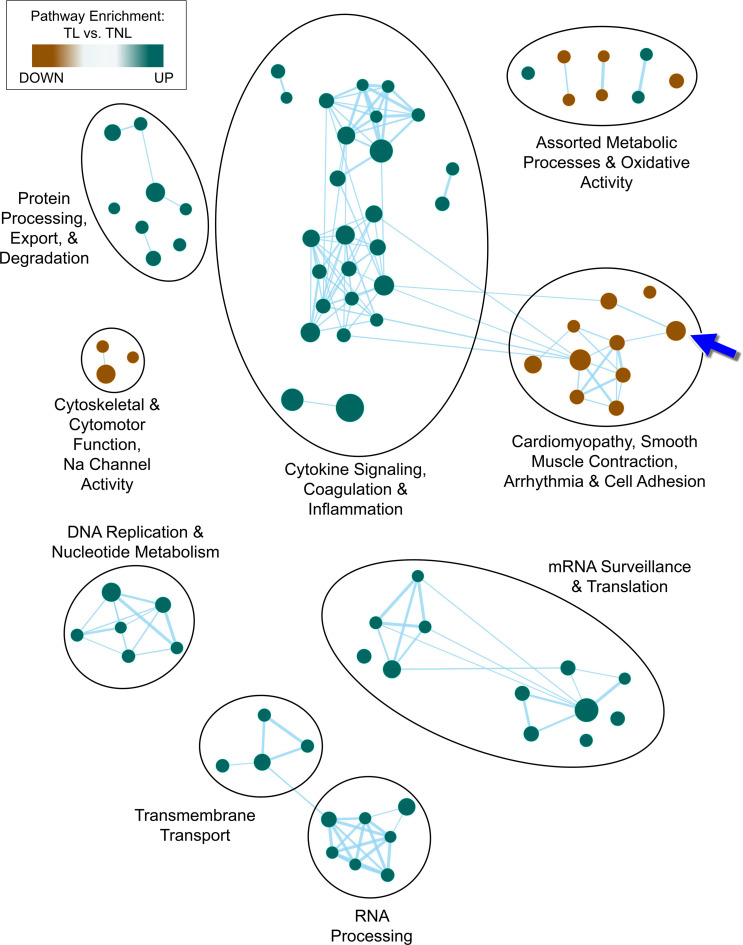
Characteristics of the transcriptional landscape differentiating the quiescent (Q) and nonquiescent (NQ) molecular phenotypes. Enrichment map of gene sets exhibiting significant enrichment by the GSEA algorithm (*P* < 0.005, FDR < 0.1) based on the transcriptional expression signature in myometrial samples. Markov cluster analysis was used to identify 9 dense subclusters within the network, indicated by the labeled ellipses. Within the network diagram, node size reflects the number of genes in each enriched set, node color indicates the direction of enrichment, and edges represent the overlap coefficient between adjacent nodes as a similarity metric. The blue arrow indicates the KEGG calcium signaling pathway within a subnetwork enriched for Ca^2+^ binding and Ca^2+^ transport genes.

To further characterize the transcriptomic signature that distinguished myometrial Q from the myometrial NQ phenotype, we applied exploratory gene set association analysis to the TL versus TNL comparison. Enrichment (FDR < 0.1) was observed for 129 individual pathways, the top 84 of which (nominal *P* < 0.005) were used for network visualization and cluster analysis. Nine general subnetworks of related gene sets were identified, with a majority of pathways relating to cytokine signaling and inflammation (26 gene sets) (Figure 3), possibly related to leukocytic infiltrate into the myometrium with labor (17). In addition to gene sets relating to various processes such as cellular maintenance, RNA processing/translation, and metabolism, we identified a subcluster of functionally related pathways (10 gene sets) comprising numerous transcripts involved in Ca^2+^ binding, Ca^2+^ transport, and calmodulin interactions. This subnetwork included the calcium signaling pathway (Figure 3, arrow) and gene sets annotated as involved in cardiomyopathy, smooth muscle contraction, cardiac conduction, and cell adhesion.

Knowledge-based inference of upstream regulatory molecules was next used to query causal pathways potentially contributing to the differential abundance of transcripts between TL and TNL myometrial specimens. Within the master regulatory network of 127 factors (Supplemental Figure 1), 23 subnetworks were identified by cluster analysis. The most statistically significant of these comprised 2 overlapping groups of nodes consisting of acute, proinflammatory innate immune signaling molecules, such as members of the NF-κB transcriptional complex (Supplemental Figure 1, left inset). Additionally, 2 closely related subnetworks of 13 and 16 interconnected nodes were distinguished, containing several proteins involved in morphogenic signaling, including members of the TGF-β and bone morphogenic protein families (Supplemental Figure 1, right inset).

**Figure 4:**
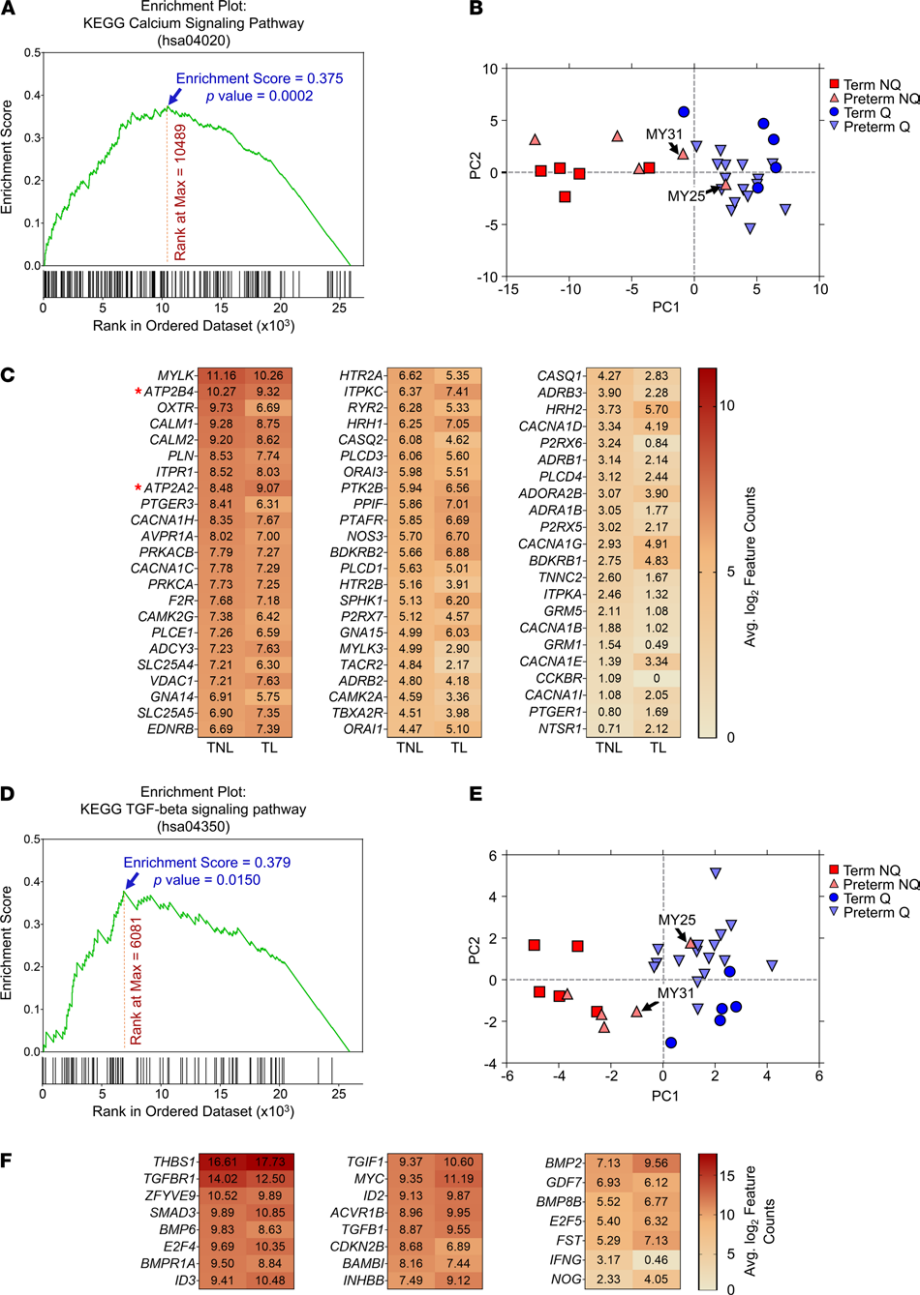
Myometrial nonquiescence is associated with potential dysregulation of Ca^2+^ signaling and TGF-β pathways. (**A**) Gene set enrichment plot for the 183 transcripts related to the KEGG calcium signaling pathway. For this analysis, gene expression ranking in the TL versus TNL comparison was done by the adjusted *P* value (FDR) as determined using the DESeq2 algorithm, and statistical enrichment for the gene set was determined using 1000 gene list permutations. (**B**) Scatterplot of the top 2 principal components following dimensionality reduction of differentially abundant calcium signaling transcripts. TNL specimens are indicated by blue circles, TL samples are indicated by red squares, and preterm NQ (nonquiescent phenotype) and Q (quiescent phenotype) specimens are depicted by light red and light blue triangles, respectively. (**C**) Heatmap of average, log_2_-transformed, normalized RNA-seq feature counts for the 68 calcium signaling transcripts exhibiting significant differences in abundance between the TL and TNL myometrial samples (FDR < 0.1, fold-change of at least ± 1.5). Asterisks denote the 2 mRNAs encoding calcium transporter proteins selected for more detailed analysis: *ATP2B4* and *ATP2A2*. Note that a difference between 2 values, *a* – *b*, in the log_2_-transformed data corresponds to a linear difference of 2*^a^* – 2*^b^*. (**D**) Gene set enrichment plot for the 84 transcripts related to the TGF-β signaling KEGG pathway, conducted as described above. (**E**) Scatterplot of the dominant principal components following PCA applied to differentially abundant TGF-β pathway transcripts. Sample annotation is equivalent to that in **B**. (**F**) Heatmap of the average normalized RNA-seq feature counts (log_2_-scaled) for the 23 TGF-β pathway genes that differed significantly between the TL and TNL myometrial samples (FDR < 0.1, minimum fold-change ± 1.5).

Given a central role for intracellular calcium in the orchestration of myometrial contractile protein activity, and in light of the functional analysis results described above, we interrogated the Ca^2+^ signaling pathway in greater detail using an unbiased list of 183 genes previously described (18). We observed significant enrichment for genes in this set within the ranked transcript list overall (*P* < 0.001) (Figure 4A). Differential abundance (FDR < 0.1 by DESeq2 analysis) was noted for 68 of these (37% of the 183 transcripts), with 44 being in greater abundance in the TNL specimens compared with the TL samples (Figure 4C). We noted that 2 of the mRNAs encoding calcium transporter proteins displayed robust expression levels throughout all samples and also exhibited anticorrelated expression patterns between Q and NQ myometrial specimens: *ATP2B4* (also known as plasma membrane calcium ATPase isoform 4, PMCA4) and *ATP2A2* (also called sarco/endoplasmic reticulum Ca^2+^ transporting 2, SERCA2) (Figure 4C, red asterisks).

We additionally analyzed the TGF-β signaling pathway with greater scrutiny, given its roles in connective tissue homeostasis and smooth muscle cell differentiation (19, 20) and given its dense clustering within the upstream regulatory network (Supplemental Figure 1). Collectively, the set of 84 TGF-β pathway genes exhibited significant enrichment (*P* < 0.05) in the TL versus TNL comparison (Figure 4D), with 23 (27%) of the pathway genes exhibiting differential expression (FDR < 0.1 by DESeq2); most of these were more abundant in TL specimens (Figure 4F).

PCA revealed strong correlations between the dominant principal components for these pathways and that of the full panel of the 4814 transcripts (*r* = 0.98 and 0.93 for the Ca^2+^ and TGF-β signaling pathways, respectively, *P* < 0.001). Either of these gene expression data subsets could distinguish the NQ from the Q specimens with almost no overlap, except for 2 preterm myometrium samples (MY25 and MY31, arrows; the latter of these was classified as NQ by the comprehensive PCA) (Figure 4, B and E).

### The expression ratio of 2 anticorrelated calcium transporter genes reliably distinguishes myometrial Q from the NQ phenotype.

**Figure 5:**
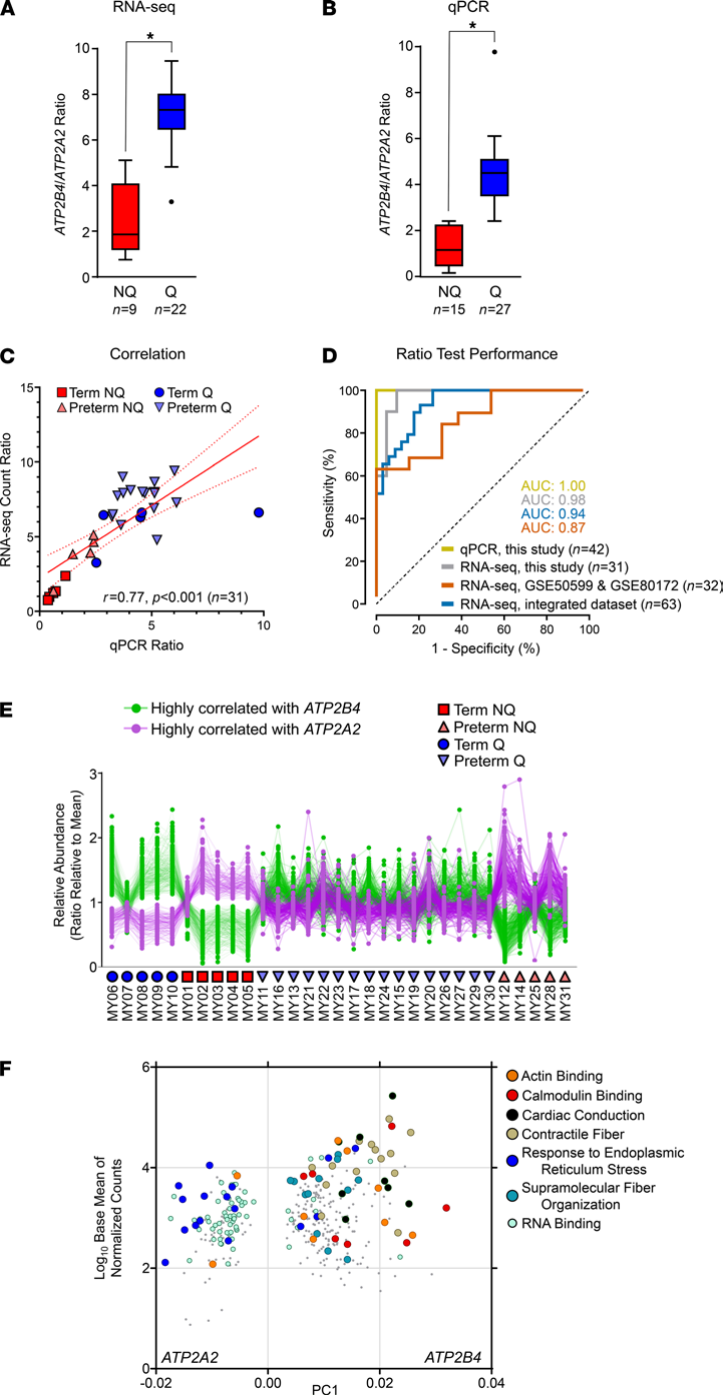
The expression ratio of 2 anticorrelated calcium transporter genes reliably distinguishes myometrial quiescence from the nonquiescent phenotype. (**A**) Box and whisker plots (box, median with IQR; whiskers, inner fences using Tukey’s method) showing expression ratios of transcripts encoding *ATP2B4* and *ATP2B2* as determined by RNA-seq (Q, quiescent phenotype, *n* = 22; NQ, nonquiescent phenotype, *n* = 9). Asterisk indicates statistical significance (*P* < 0.001, Mann-Whitney *U* test). (**B**) Box and whisker plots (as in **A**) showing qPCR expression ratios of *ATP2B4*/*ATP2B2*, stratified by phenotype (Q, *n* = 27; NQ, *n* = 15). Asterisk indicates statistical significance (*P* < 0.001, Mann-Whitney *U* test). (**C**) Scatterplot showing the extent of correlation between *ATP2B4*/*ATP2B2* expression ratios determined using RNA-seq and qPCR in 31 samples (*r* = 0.76, *P* < 0.001). TNL specimens are indicated by blue circles, TL samples are indicated by red squares, and preterm NQ and Q specimens are depicted by light red and light blue triangles, respectively. (**D**) ROC curve analysis applied to binary classification of NQ and Q specimens based on expression ratios of *ATP2B4*/*ATP2B2* calculated either by qPCR (for samples from the current study) or RNA-seq (for samples from the current study, samples from prior published studies [GSE50599, *n* = 10; and GSE80172, *n* = 22], and an integrated data set comprising samples from the current study and the 2 existing data sets). All AUC values achieved statistical significance (*P* < 0.001). (**E**) Relative abundances of transcripts with expression highly correlated (*r* ≥ 0.95) with *ATP2B4* (233 transcripts, green) or *ATP2A2* (121 transcripts, purple). TNL specimens are indicated by blue circles, TL samples are indicated by red squares, and preterm NQ and Q specimens are depicted by light red and light blue triangles, respectively. (**F**) Overrepresented pathways for transcripts in **E**, plotted by average log_10_-scaled baseline expression in Q (term and preterm) specimens, and projection of transcript along PC1 in principle component analysis.

When the feature counts for *ATP2B4* and *ATP2A2* transcripts in the RNA-seq data set were stratified by myometrial phenotype (Q versus NQ), their ratio was significantly diminished in specimens with clinically identifiable myometrial contractions (*P* < 0.001, Mann-Whitney *U* test, *n* = 31 specimens; Figure 5A). To cross-validate and extend these results, we next evaluated the expression of these transcripts using quantitative PCR (qPCR) in an expanded set of myometrial samples. By this method, we found that the ratio of *ATP2B4*/*ATP2A2* was 3.5-fold lower on average in clinically contractile specimens (*P* < 0.001, Mann-Whitney *U* test, *n* = 42 specimens, including 11 specimens used for the qPCR analysis only; Figure 5B). Similar statistically significant results (*P* < 0.01, Mann-Whitney *U* test) were obtained when the 11 validation cohort specimens were considered separately. The correlation between the corresponding qPCR and RNA-seq ratios was *r* = 0.77 (*P* < 0.001) for the 31 specimens for which paired data were available (Figure 5C).

We next evaluated potentially confounding effects of type of uterine incision/region of biopsy collection (fundal versus lower uterine segment) on *ATP2B4*/*ATP2A2* ratios. Among PTB cases, the proportion delivered by classical cesarean section did not differ from those delivered via a low transverse uterine incision when stratified by molecular phenotype (*P* = 0.343, Fisher’s exact test), nor were the *ATP2B4*/*ATP2A2* ratios significantly different when grouped by uterine incision type, as determined using either qPCR or RNA-seq (*P* = 0.411 and *P* = 0.956, respectively, Mann-Whitney *U* test). Type of uterine incision was not significantly correlated with the contractile state of the myometrium (*r* = 0.23, *P* = 0.301). In regression models, inclusion of uterine incision type as a covariate or institutional site of patient recruitment did not change the significance of the association between *ATP2B4*/*ATP2A2* ratio and uterine molecular phenotype (Q versus NQ).

In our specimens, receiver operating characteristic (ROC) curve analysis of the *ATP2B4*/*ATP2A2* ratio by qPCR yielded an AUC of 1.00; all NQ specimens had ratios ≤ 2.41 (Figure 5D). ROC analysis for the corresponding RNA-seq–based *ATP2B4*/*ATP2A2* ratio in our data set revealed an AUC of 0.98, with 2 of 31 samples (7%) being misclassified using an NQ threshold ≤ 5.12 (Figure 5D).

To assess the utility of the RNA-seq–based classification system across studies, we also abstracted the *ATP2B4*/*ATP2A2* ratio data from 2 existing myometrial sample RNA-seq data sets — GSE50599 (16) and GSE80172 (13) — using the same analytical pipeline that was used to evaluate the present RNA-seq data. These data sets only include samples classified clinically at term, since — before the current study (to the best of our knowledge) — no publicly accessible RNA-seq data sets from preterm myometrium were available. Using a binary classification system (all samples were classified as TL or TNL based on provided information), ROC analysis of these additional data sets resulted in AUCs of 0.79, 1.00, and 0.87 for the GSE80172 data, the GSE50599 data set, and the combination of these, respectively (Figure 5D). Integration of the 2 existing data sets with the RNA-seq ratio data from the present study revealed an overall AUC of 0.94 (Figure 5D).

To assist future investigations, we additionally collated a list of genes with RNA-seq expression patterns having the highest degree of correlation (*r* ≥ 0.95) with either *ATP2B4* or *ATP2A2*, given that these genes might also be suitable for use in NQ versus Q molecular stratification and may play a functional role pending further study (Figure 5E and Supplemental Table 4). Transcripts highly correlated with *ATP2B4* expression were enriched for actin and calmodulin binding, cardiac conduction, and contractile fiber pathways, while those correlated with *ATP2A2* expression had overrepresentation for genes involved in endoplasmic reticulum stress response, supramolecular fiber organization, and RNA binding activity (Figure 5F).

## Discussion

While TL (1) is straightforward to diagnose in the presence of uterine contractions accompanied by progressive cervical dilatation at an advanced GA, recognition of pregnancies destined to deliver preterm can be misleading based on clinical presentation alone (4, 21). In practice, PTB is a complex phenotype, in which significant maternal, fetal, and placental conditions may individually or collaboratively contribute to spontaneous labor initiation, as manifest through one or more of the above common pathway events and/or vaginal bleeding (6, 7). Importantly, and in contrast to the typical presentation of term parturition, the PTB-associated phenotype of the uterine corpus might diverge from that of the uterine cervix, implying regional variability in the response of this organ to initiating events.

In the present study, we posited that the laboring myometrium displays a characteristic contraction-associated transcriptomic signature that may be inconsistently manifest across samples identified using clinical criteria alone. To establish a molecular signature distinguishing the NQ state, we began by cataloging myometrial transcripts that were differentially abundant within term samples delivered in unambiguous instances of myometrial activation and contractility. This yielded a candidate panel of 4814 transcripts characterized by genes involved in immune signaling, inflammation, contractility, morphogenic signaling, metabolism, and redox activity, among others. Dimensionality reduction based on these transcripts resulted in segregation of term specimens into 2 independent groups along the major principal component axis, coinciding with the absence or presence of myometrial contractility. When applied to the PTB specimens, this functional genomics approach revealed a small subset of cases in which myometrial contractions were identified clinically prior to delivery, indicating mismatches between clinical phenotyping and molecular classification of preterm myometrial tissues. Based on these results, we proceeded to examine whether a more limited panel of genes might be suitable for molecular classification. To this end, we surveyed biological pathways potentially dysregulated in the setting of TL as a framework, ultimately prioritizing differentially abundant transcripts of the Ca^2+^ and TGF-β signaling pathways as potentially important components of the general mechanisms of labor initiation. PCA revealed strong correlations between the dominant principal components for these pathways and those of the full panel of the 4814 transcripts, suggesting that either subset could be employed for molecular classification of the myometrial state.

Given these strong correlations, we next examined whether we could further reduce pathway-based stratification to a limited number of Ca^2+^ signaling genes. We cross-validated a pair of calcium-handling transcripts that (a) exhibited reciprocal expression patterns in the setting of TL; (b) coded for proteins that regulate smooth muscle contractility by reducing intracellular Ca^2+^ levels following stimulation (22); and (c) were the predominant contributors to PCA-based stratification among genes of the Ca^2+^ signaling pathway. Uterine contractility was associated with diminished expression of the plasma membrane pump *ATP2B4*. Transcripts highly correlated with *ATP2B4* expression were enriched for calmodulin binding, contractile fiber organization, and ion channel activities. In addition, contractility increased abundance of the endomembranous transporter *ATP2A2*, which directs cytoplasmic Ca^2+^ to the sarco/endoplasmic reticulum lumen and contributes to the regulation of the contraction/relaxation cycle in muscle tissues (23, 24). In the laboring uterus, the increased expression and activity of SERCA2 (the cognate *ATP2A2* mRNA product) is thought to play a role in frequency and duration of spontaneous contractions (25). Transcripts highly correlated with *ATP2A2* expression included those involved in the endoplasmic reticulum stress response, a pathway that was previously proposed to orchestrate myometrial Q via nonapoptotic caspase 3 and/or inhibition of local inflammatory signaling (26–28). Using qPCR in an expanded set of specimens, we found that the ratio of *ATP2B4*/*ATP2A2* could reliably classify the myometrial phenotype in both term and preterm cases with a high degree of accuracy.

To assess the utility of the *ATP2B4*/*ATP2A2* ratio in myometrial phenotype calling more generally, we next evaluated its performance by integrating the current RNA-seq data with that from 2 existing data sets: GSE50599 and GSE80172. In these specimens, *ATP2B4*/*ATP2A2* ratios ranged from to 0.75 to 9.43, with higher values indicative of myometrial Q. Using a threshold ratio of < 5.13 to assign NQ, our results show that the overall classification accuracy was high. We found that a majority of misclassifications occurred for midrange ratio values (Supplemental Figure 2) and were most frequent for specimens from the GSE80172 data set. In their work employing statistical learning strategies to transcriptomics-based myometrial phenotyping, Stanfield and colleagues previously noted consistent misclassification of several GSE80172 samples (13). Although sample misidentification (e.g., through clerical errors during sample processing and analysis) cannot be excluded in these instances, it is noteworthy in light of the present data that classification problems were most frequent for marginal *ATP2B4*/*ATP2A2* ratios. From this perspective, strict binary classification strategies might be unrealistic for the purposes of myometrial phenotyping, and indeed, choosing a singular threshold for categorization presents something of a demarcation dilemma. Instead, it might be more appropriate to consider classification strategies that include a “transitional” category between the more extreme phenotypes of unambiguous myometrial Q and NQ.

At present, it is difficult to implement the knowledge derived from our findings in routine clinical practice. However, some inferences of clinical significance could be made. For example, it is possible that tocolytics could be of benefit only if a certain level of activation at the molecular level is present. However, as seen in our study, clinical features of labor are imperfect in detecting the molecular contractile phenotype, and a transition state may also exist. Future investigations of noninvasive biomarkers could focus on detecting the molecular myometrial state based on the *ATP2B4*/*ATP2A2* ratio and/or correlated transcripts reported in this study.

Our study has several limitations. This is because establishing Q/NQ/transitional status was possible only through analysis of myometrial biopsies retrieved through a cesarean delivery due to various conditions. As such, sampling could not be performed on patients who ultimately completed normal vaginal delivery. As with prior functional genomic studies of term myometrium, the clinical conditions necessitating cesarean delivery (e.g., labor dystocia or fetal intolerance of labor) raise concerns about the generalizability of findings to populations undergoing physiological TL (29, 30). In the current study, internal tocodynamometry data were available for term laboring specimens to verify myometrial contractility and to help guide molecular classification, but this information was not available in preterm deliveries. Second, a number of the specimens were delivered following administration of oxytocin for induction or augmentation of labor. While it remains to be elucidated the extent to which such treatments might affect global myometrial gene expression, a previous sensitivity analysis by Sharp and colleagues found that pharmaceutical labor initiation or augmentation was not a large confounding factor in myometrial gene expression profiling (29). Additionally, we considered that differences in the location of uterine sampling (following either low transverse or classical uterine incisions at time of cesarean) might have contributed, to some extent, to the observed biomolecular changes we have reported (15, 31, 32). However, the type of incision and the site of the biopsy did not significantly contribute to changes in *ATP2B4*/*ATP2A2* ratios. As far as we are aware, our study provides the largest myometrial RNA-seq data set to date that, together with the granular clinical metadata, could serve as a starting point for additional analyses by others and for future targeted mechanistic investigations.

In conclusion, studies designed to increase understanding of the mechanistic determinants underlying the transition from uterine Q to labor in humans typically rely on specimens categorized by clinical signs and symptoms. While indispensable for phenotypic classification, these metrics may be subjective and imprecise. In this study, we provide a more objective means of classifying myometrial specimens along the continuum between Q and contractility using the expression of just 2 calcium transporter genes. We submit that such molecular phenotyping might provide a quality control metric similar to those routinely employed in genomics research (33). This procedure could mitigate misclassification of the myometrial phenotype based on the false assumption that a pregnancy ending in PTB due to PPROM, advanced cervical dilation, or Triple I must also be associated with a laboring myometrium. Additional work will be necessary to ascertain whether molecular signatures such as those presently observed in the myometrium may be reflected in other compartments — particularly in the maternal circulation.

## Methods

### Patients and myometrial biopsies

The study included myometrial biopsies from 42 individuals pregnant with singletons as described in further detail below.

#### Exploratory phase.

This first stage of the study involved biopsies from 31 patients undergoing primary cesarean deliveries. Cases were carefully phenotyped with respect to GA, circumstances of labor onset, and clinical status at the start and end of the intervention. Case aggregation was as described in Results. Additional phenotyping of cases spontaneously committed to PTBs (Group 3 and Group 4) involved presence or absence of Triple I based on analysis of amniotic fluid obtained by clinically indicated transabdominal amniocentesis. Amniotic fluid was analyzed for glucose concentration, lactate dehydrogenase activity, WBC and RBC count, Gram stain, and microbial cultures including for *Ureaplasma* and *Mycoplasma* spp. as described in prior studies (34–36). Additionally, placentas from all PTB cases were reviewed by clinical pathologist to evaluate for the presence of histological chorioamnionitis.

#### Validation stage.

The second stage of the study was intended to confirm select transcripts by qPCR and included myometrial biopsies from 11 additional cases (2 preterm and 9 term), of which 6 were primary and 5 were repeat cesareans. All 19 term cesareans were performed via lower uterine segment incision. Among preterm cesareans, 61% (14 of 23) of uterine incisions were vertical. A detailed description of clinical definitions employed in this study is included in the supplemental material.

All myometrial specimens were collected immediately after delivery of the fetus and placenta by a single investigator. The biopsies consisted of a full-thickness strip of myometrial tissue from the upper lip of the incision in low-transverse cesarean sections or the side at the midpoint of the incision in classical vertical incisions. A portion of the tissue biopsy free of decidua and serosa was immediately snap frozen in liquid nitrogen and stored at –80°C.

### RNA-seq and analysis

Total RNA was extracted from all biopsy specimens using TRIzol reagent (Invitrogen) and processed as previously reported (36, 37). All samples submitted for sequencing had an RNA integrity number (RIN) ≥ 6. rRNA-depleted sequencing libraries were constructed using the TruSeq Stranded Total RNA Sample Prep Kit with Ribo-Zero Gold (Illumina) and were sequenced on the Illumina HiSeq 2500 platform to generate 50 bp single-end reads at a target depth of 30 million. Read quality was assessed using FastQC, and adaptor and quality trimming was achieved with Trim Galore using the default settings. Reads were then mapped to the hg38 genome assembly using TopHat2 (38), and feature counts were generated using HTseq (39). Differential expression was statistically determined with DESeq2 (40) using an absolute linear fold-change cut-off ≥ 1.5 and FDR < 0.1 (41). As the sequenced reads were generated in 2 separate batches, statistical models were generated both without and with inclusion of batch as covariable. In the case of the TNL versus TL comparison, all samples were sequenced in the same batch, so the batch effect could not be fit with modeling and was omitted; however, batch of origin was included in the statistical modeling of the PTB samples. Two of the term RNA samples (MY05 and MY08) were subjected to library preparation and sequencing in both batches, and the resulting mapped feature counts were found to be highly correlated (*r* = 0.998 for both).

### qPCR

For cross-validation studies, we selected sarco/endoplasmic reticulum calcium ATPase 2 (SERCA2, encoded by *ATP2A2*) and plasma membrane calcium-transporting ATPase 4 (PMCA4, encoded by *ATP2B4*). Reverse transcription for these reactions was performed with Superscript II Reverse Transcriptase (Invitrogen) using oligo(dT) primers. The following TaqMan gene expression assays (Thermo Fisher Scientific) were used: Hs00544877 (*ATP2A2*) and Hs00608066 (*ATP2B4*). The geometric mean of the Ct values for β-2 microglobulin (*B2M*, catalog 4331182) and ribosomal protein L30 (*RPL30*, catalog 4331182) was used as a reference in each reaction. Each 20 μL reaction consisted of 1 μL cDNA (500 ng), 1 μL of TaqMan Gene Expression Assay, 10 μL TaqMan Fast Advanced Master Mix (Thermo Fisher Scientific), and 8 μL of nuclease free water. All reactions were performed in duplicate. The relative abundance of each mRNA was calculated using comparative Ct method (42), and statistical testing was performed using Prism software (GraphPad Software). The ratio of *ATP2B4*/*ATP2A2* was calculated as 2^–ΔC^, with ΔC representing the difference between the Ct values for *ATP2B4* and *ATP2A2* (i.e., ΔC = Ct*^ATP2B4^* – Ct*^ATP2A2^*).

### Pathway analysis

Gene set enrichment analysis (GSEA) was performed using the GSEA desktop application using the signal-to-noise gene list ranking metric and execution of 1000 permutations for gene sets between 15 and 500 members in size (43). Networks were generated in the Cytoscape (44) software environment with the Enrichment Map (45) plugin, using an uncorrected *P* value threshold of 0.005, an FDR cutoff of 0.1, and an overlap coefficient (i.e., the size of the intersection between 2 adjacent data sets divided by the size of the smaller of 2 data sets being compared) threshold of 0.1. Markov cluster analysis was then used to identify dense subclusters within the network using the AutoAnnotate (46) application, with Jaccard similarity indices comprising edges between adjacent gene network nodes. Additionally, Ingenuity Pathways Analysis (IPA; Qiagen) was used to infer biologically plausible upstream regulatory and signaling events potentially contributing to observed differential gene expression signatures (47). IPA results were filtered by molecule type to include enzymes, cytokines, growth factors, transcription regulators (including ligand-dependent nuclear receptors), translation regulators, transmembrane receptors/transporters/ion channels, protein kinases, and phosphatases. Network diagrams were rendered using Cytoscape and evaluated using the ClusterONE plugin (48).

### Data deposition and comparison with publicly available RNA-seq data sets

The RNA-seq data presented in this publication has been deposited in the Gene Expression Omnibus (GEO) database (https://www.ncbi.nlm.nih.gov/geo/) and is accessible through GEO Series accession no. GSE163773. To contextualize the current study considering prior work, we abstracted RNA-seq data from 2 existing term myometrial cohorts available through GEO: GSE50599 (16) and GSE80172 (13). The GSE50599 data set was from a transcriptomics analysis of term myometrial specimens in the absence (*n* = 5) or presence (*n* = 5) of labor, while the GSE80172 cohort was classified into 3 categories based on TL stage: not in labor (*n* = 8), initial or early labor (defined as regular uterine contractions with cervical dilatation < 3 cm, *n* = 8), and established labor (defined as regular uterine contractions with cervical dilatation > 3 cm, *n* = 6). The same analytical procedure described above was also used for mapping and feature count generation for these files.

### Statistics

Pearson correlations were calculated using the cor() function in the base R package. PCA for differentially expressed transcripts was accomplished using the prcomp() function in base R, in addition to the factoextra package (49). ROC curve analysis was performed using MedCalc (MedCalc Software Ltd.). Additional statistical testing and graphs were made using GraphPad Prism and MedCalc (MedCalc Software Ltd.) software. Except where otherwise noted, *P* <0.05 was considered statistically significant.

### Study approval

This study was approved by the IRBs of Yale University, The Ohio State University, The Research Institute at Nationwide Children’s Hospital, and University of Illinois at Chicago. All study participants provided written informed consent.

## Author contributions

WEA, IAB, and CSB had the idea and developed the study design. WEA, GZ, and TLS executed the RNA extraction and qPCR experiments. WEA, AS, and IAB conducted the data analysis with input from CSB. CSB participated with patient recruitment, abstraction of clinical data, and assisting with aspects of the study design. WEA, CSB, and IAB wrote the first draft of the manuscript. All authors participated with reviewing the findings of the work and contributed with critical revisions to the drafts of the manuscript.

## Supplementary Material

Supplemental data

Supplemental Table 1

Supplemental Table 2

Supplemental Table 3

Supplemental Table 4

## Figures and Tables

**Table 1:**
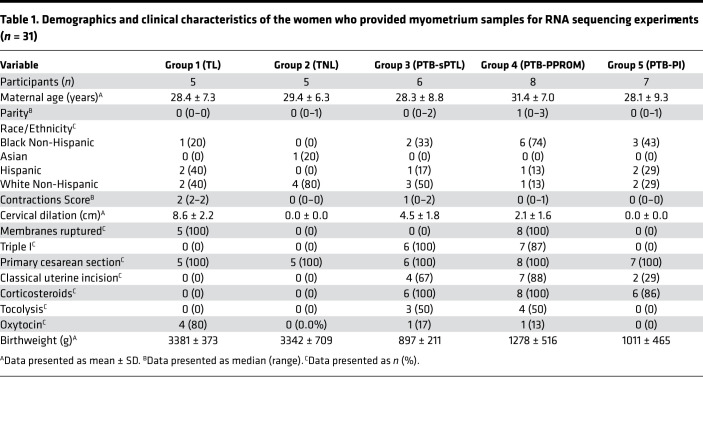
Demographics and clinical characteristics of the women who provided myometrium samples for RNA sequencing experiments (*n* = 31)

## Version 1 05/04/2021

In-Press Preview

## Version 2 06/08/2021

Electronic publication
