# Supplementary material for: Molecular signatures of labor and nonlabor myometrium with parsimonious classification from 2 calcium transporter genes
Source: JCI Insight. 2021 Jun 8;6(11):e148425. doi: 10.1172/jci.insight.148425 (PMC8262336; doi:10.1172/jci.insight.148425)
Supplement: Supplemental data [file jciinsight-6-148425-s023.pdf]

SUPPLEMENTAL MATERIAL

Molecular Phenotype Signatures of Labor and Non-Labor Human Myometrium with Parsimonious Classification from Two Calcium Transporter Genes

William E. Ackerman IV[1], Catalin S. Buhimschi[1], Ali Snedden[2], Taryn L. Summerfield[3] Guomao Zhao[1], Irina A. Buhimschi[1]

Affiliations:

[1]Department of Obstetrics and Gynecology, University of Illinois at Chicago College of Medicine, Chicago, IL 60612, USA

[2]The High Performance Computing Facility, The Research Institute at Nationwide Children's Hospital, Nationwide Children's Hospital, Columbus, OH 43215, USA

[3]The Ohio State University College of Medicine, Department of Obstetrics & Gynecology, Columbus, OH 43212, USA

This supplementary material along with the raw data files listed herein are provided by the authors for additional detail regarding the present study and to aid others in their own work.

TABLE OF CONTENTS

A. SUPPLEMENTAL METHODS

Inclusion and exclusion criteria.

Clinical definitions.

Semi-quantitative clinical phenotype classification algorithm.

B. SUPPLEMENTAL TABLES

Supplemental Table S1. Detailed clinical characteristics of myometrial sample donors.

Supplemental Table S2. Transcripts differentially abundant between term labor (TL) and term non-labor (TNL) myometrial biopsy specimens.

Supplemental Table S3. Transcripts differentially abundant between non-quiescent (NQ) and quiescent (Q) preterm myometrial biopsy specimens.

Supplemental Table S4. Transcripts with expression most highly correlated with that of *ATP2B4* or *ATP2A2*.

C. SUPPLEMENTAL FIGURES

Supplemental Figure S1. Upstream regulator network inferred from the myometrial transcriptional expression patterns differentiating the quiescent (Q) and non-quiescent (NQ) molecular phenotypes.

Supplemental Figure S2. Details of classification accuracy for *ATP2B4*/*ATP2A2* RNA-seq expression ratio across three studies.

A. SUPPLEMENTAL METHODS

Inclusion and exclusion criteria.

All patients had a singleton fetus and had requested epidural or combined spinal-epidural analgesia in anticipation for Cesarean delivery. Inclusion criteria for term laboring (TL) women required regular myometrial contractions and ≥4 cm cervical dilation. At term, TL women met the criteria for labor dystocia (failure to descent or progress). Preterm patients were undergoing Cesarean section for a variety of indications as noted in Supplemental Table S1.

Exclusion criteria included suspected fetal macrosomia (4500 g by either clinical or ultrasonographic evaluation), abnormalities of placentation requiring emergent Cesarean section (low-lying placenta, placental abruption) and uterine structural abnormalities. A prior uterine scar was an exclusion criterion for samples subjected to RNA sequencing which were all primary Cesareans.

Clinical definitions.

Gestational age (GA) was established based on last menstrual period confirmed by an ultrasonographic examination prior to 20 weeks of gestation (1). Triple I (intra-amniotic infection and/or inflammation) was established based on analysis of the amniotic fluid retrieved in sterile conditions by trans-abdominal amniocentesis (2). Amniotic fluid infection was established by a positive Gram stain and/or a positive microbial culture result. The diagnosis of histological chorioamnionitis was established through histologic examination for inflammation of the placenta and fetal membranes, which was assessed by a clinical pathologist (3). Preterm birth was defined as delivery of the neonate <37 weeks GA (4). Idiopathic preterm birth was established in the absence of Triple I or histologic chorioamnionitis as assessed by a clinical pathologist (5). Preeclampsia and severity thereof was established using clinical criteria established by the American College of Obstetricians and Gynecologists’ Task Force on Hypertension in Pregnancy (6).

Semiquantitative phenotypic classification algorithm.

Gestational age was dichotomized into term (1: delivery >37 weeks of gestation) and preterm (0: delivery <37 weeks of gestation) cases. Uterine contractions were scored semiquantitatively on a scale from 0-2 (0: absent; 1: irregular, not followed by cervical change or when contractions receded after tocolysis; or 2: regular and followed by cervical change). Cervical dilation was scored on a scale from 0-10 cm as recorded on the last exam prior to Cesarean. Membrane status was scored as 0 (intact) or 1 (ruptured). Triple I was scored based on amniocentesis results as 0 (absent) or 1 (confirmed).

B. SUPPLEMENTAL TABLES

SUPPLEMENTAL TABLE S1. Detailed demographic and clinical characteristics of myometrial sample donors.
File: Table S1.xlsx

SUPPLEMENTAL TABLE S2. Transcripts differentially abundant between term labor (TL) and term non-labor (TNL) myometrial biopsy specimens.
File: Table S2.xlsx

SUPPLEMENTAL TABLE S3. Transcripts differentially abundant between non-quiescent (NQ) and quiescent (Q) preterm myometrial biopsy specimens.
File: Table S3.xlsx

SUPPLEMENTAL TABLE S4. Transcripts with expression most highly correlated with that of *ATP2B4* or *ATP2A2*.
File: Table S4.xlsx

C. SUPPLEMENTAL FIGURES

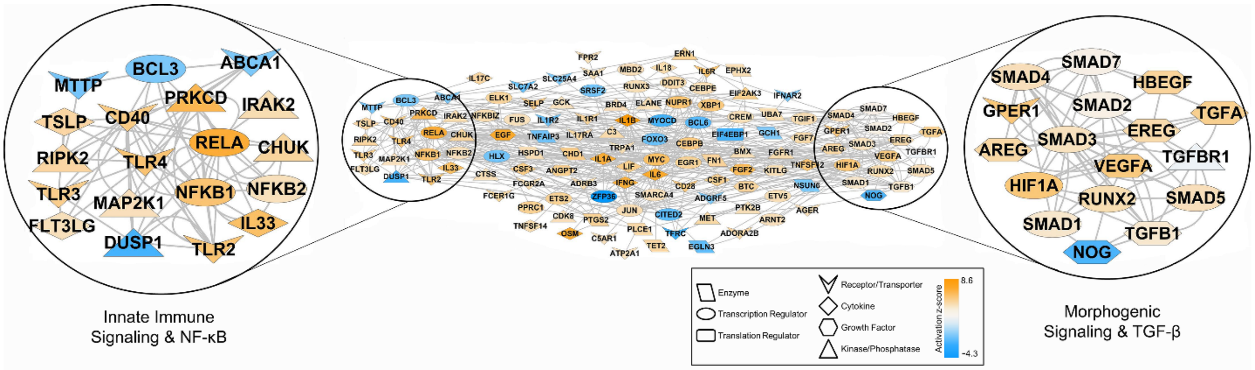

SUPPLEMENTAL FIGURE S1. Upstream regulator network inferred from the myometrial transcriptional expression patterns differentiating the quiescent (Q) and non-quiescent (NQ) molecular phenotypes. Interaction network of 127 factors identified by the IPA upstream regulator causal inference algorithm based on the 4,184-transcript expression signature in myometrial samples, rendered using Cytoscape. IPA results were filtered by molecule type to include enzymes, cytokines, growth factors, transcription regulators (including ligand-dependent nuclear receptors), translation regulators, transmembrane receptors/transporters/ion channels, protein kinases, and phosphatases. Within the master regulatory network, two overlapping, densely connected subclusters comprising acute, pro-inflammatory innate immune signaling molecules were identified using the ClusterONE algorithm (left inset). Additionally, the ClusterONE algorithm detected two closely related subnetworks containing regulators involved in morphogenic signaling, including members of the transforming growth factor and bone morphogenic protein families (right inset).

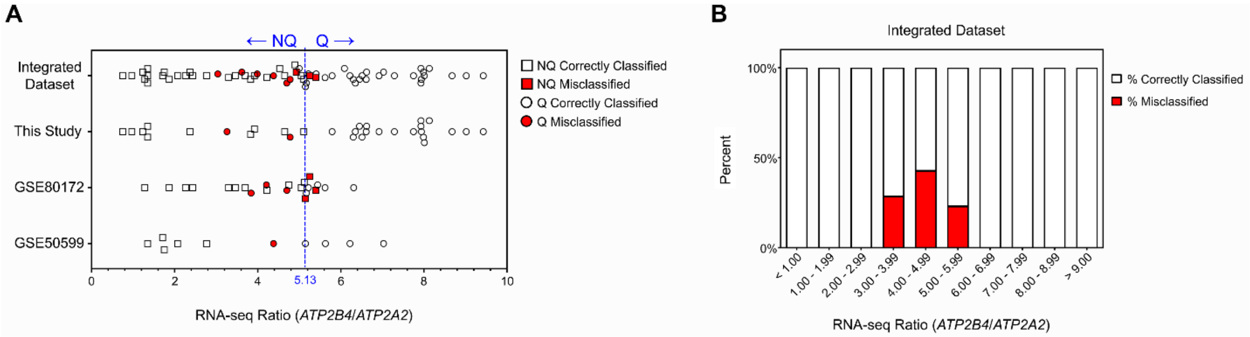

SUPPLEMENTAL FIGURE S2. Details of classification accuracy for *ATP2B4*/*ATP2A2* RNA-seq expression ratio across three studies. (A) Scatterplot of individual *ATP2B4*/*ATP2A2* RNA-seq ratio vales shown in aggregate, and in three studies individually: GSE80172, GSE50599, and the current study. Classification using a threshold ratio of 5.13 to distinguish functional non-quiescence (NQ, lower values; square symbols) from quiescence (Q, higher values; circles) resulted in misclassification (red symbols) of 9/63 (14%) of samples overall (sensitivity: 90%; specificity: 82%). The two misclassified samples in the current study were MY07 and MY20. (B) Stacked bar graphs summarizing the extent of misclassification expressed as percent of total cases for each bin based on the *ATP2B4*/*ATP2A2* expression ratio determined using RNA-seq data from the current dataset and two existing datasets (GSE50599 and GSE80172).

SUPPLEMENTAL REFERENCES

1. Committee Opinion No 700: Methods for Estimating the Due Date. *Obstet Gynecol*. 2017;129(5):e150-e154.

2. Higgins RD, Saade G, Polin RA, et al. Evaluation and Management of Women and Newborns With a Maternal Diagnosis of Chorioamnionitis: Summary of a Workshop. *Obstet Gynecol*. 2016;127(3):426-436.

3. Salafia CM, Weigl C, Silberman L. The prevalence and distribution of acute placental inflammation in uncomplicated term pregnancies. *Obstet Gynecol*. 1989;73(3 Pt 1):383-389.

4. American College of Obstetricians and Gynecologists, Committee on Practice Bulletins-Obstetrics. ACOG practice bulletin no. 127: Management of preterm labor. *Obstet Gynecol*. 2012;119(6):1308-1317.

5. Buhimschi IA, Zhao G, Rosenberg VA, Abdel-Razeq S, Thung S, Buhimschi CS. 2315798; Multidimensional proteomics analysis of amniotic fluid to provide insight into the mechanisms of idiopathic preterm birth. *PLoS One*. 2008;3(4):e2049.

6. Hypertension in pregnancy. Report of the American College of Obstetricians and Gynecologists' Task Force on Hypertension in Pregnancy. *Obstet Gynecol*. 2013;122(5):1122-1131.
